# Relationships between plasma levels and six proinflammatory interleukins and body composition using a new magnetic resonance imaging voxel-based technique

**DOI:** 10.1016/j.cytox.2020.100050

**Published:** 2020-12-21

**Authors:** Robin Strand, Joel Kullberg, Håkan Ahlström, Lars Lind

**Affiliations:** a Division of Radiology, Department of Surgical Sciences, Uppsala University, Uppsala, Sweden; b Department of Information Technology, Uppsala University, Uppsala, Sweden; c Antaros Medical AB, BioVenture Hub, Mölndal, Sweden; d Department of Medical Sciences, Uppsala University, Uppsala, Sweden

**Keywords:** Whole-body imaging, Interleukins, Correlations maps, Magnetic Resonance Imaging, Imiomics

## Abstract

**Background:**

Obesity has previously been linked to inflammation. Here we investigated how plasma levels of six interleukins were related to body fat distribution.

**Methods:**

In 321 subjects, all aged 50 years, in the population-based POEM study (mean BMI 26–27 kg/m^2^), six interleukins were measured together with a DXA scan for determination of fat and lean mass. Also a whole-body magnetic resonance imaging (MRI) scan, in which fat content measurements were acquired in > 1 million voxels was performed. Interleukin levels were related to each of these voxels by the voxel-based technique “Imiomics” to create a 3D-view of how these measurements were related to size of each part of the body.

**Results:**

Levels of IL-1RA and IL-6 were related to traditional DXA and MRI measurements of adipose tissue at all locations. Neither IL-6R, nor IL-8 or IL-18, showed any consistent significant relationships vs the traditional measurements of body composition, while IL-16 showed relationships being of borderline significance. The Imiomics evaluation further strengthen the view that IL-1RA and IL-6 were related to subcutaneous adipose tissue (SAT), as well to ectopic fat distribution. In women, IL-16 levels were weakly related to expansion of SAT in the upper part of the body, while on the contrary, IL-8 levels were related to a reduction of SAT volume.

**Conclusion:**

Of the six evaluated interleukins, plasma IL-1RA and IL-6 levels were related to the amount of adipose tissue in all parts of the body, while a diverse picture was seen for other interleukins, suggesting that different interleukins are related to fat distribution in different ways.

## Highlights

• IL-1RA and IL-6 levels were related to traditional DXA and MRI measurements of adipose tissue.
• Neither IL-6R nor IL-8 or IL-18 showed strong relationships vs the traditional measurements.
• Weak relationships between IL-16 levels and trunk SAT volume was found by Imiomics.
• On the contrary, IL-8 levels were related to a reduction of SAT volume.

## Graphical abstract

**Figure:**
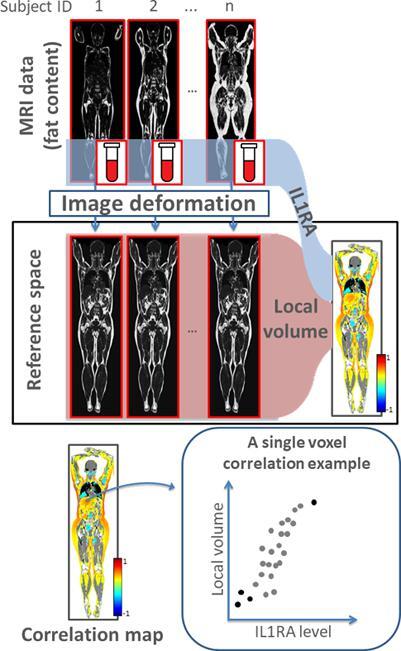

## 1 Introduction

Obesity has since>20 years been identified as a low-grade proinflammatory state. This was mainly based on the observations that C-reactive protein (CPR) and interleukin-6 (IL-6) levels were increased in obesity [1], [2], [3], and that weight loss was associated with a decline in cytokine levels [4], [5].

Adipose tissue macrophages (ATM) are a pivotal part of both SAT and VAT, and are key to the association between interleukins and body composition. As recently reviewed by Li et al. [6], the ATM are derived from circulating monocytes by monocyte chemotactic protein 1 (MCP-1). Proinflammatory ATMs (M1) are known to secrete a number of proinflammatory cytokines, as measured in the present study. Anti-inflammatory ATMs (M2) are mainly found in lean subjects. Several subtypes of ATMs have been identified. For example, some are removing dead adipocyte debris, while others are filled with lipids and secrete exosomes. These different types are producing different kinds of proinflammatory cytokines. Thus, the composition of different ATMs in different fat depots seem to be governing the expression of different interleukins, as reviewed by Engin [7]. According to the tissue expression data database GTEx (https://gtexportal.org/home/), all of the proinflammatory interleukins evaluated in the present study were expressed in human adipose tissue, with generally higher expression in VAT compared to SAT.

It was early suggested that abdominal obesity was the main driver of the relationship between obesity and inflammation [8], and experimental studies showing that visceral adipose tissue (VAT) produced more proinflammatory cytokines than subcutaneous adipose tissue (SAT) supported that suggestion [9], [10], [11]. However, when the amount of VAT and SAT have been measured by computerized tomography (CT), or by magnetic resonance imaging (MRI), some studies suggest that IL-6 levels are more strongly linked to VAT than to other indices of obesity, while others could not confirm that finding [12], [13], [14], [15], [16], [17], [18], [19].

Most studies investigating the role of inflammation in obesity have used CRP and/or IL-6. However, many other interleukins exist with important effect in the immune system, and circulating levels of also other interleukins, such as IL-8, IL-16, IL-18 or IL-1/IL-1RA have been associated with obesity [5], [20], [21], [22], [23]. However, the links to ectopic fat distribution have generally not been studied in detail for those interleukins.

Another facet of ectopic fat accumulation than VAT is increased liver fat. Although inflammation is a suggested feature of non-alcoholic fatty liver disease (NAFLD), data on circulating levels of interleukins in NAFLD are rather scarce, but increased levels of IL-6 and IL-8 [24], [25], but not IL-18 [26], have been reported in individuals with NAFDL.

The aim of the present study is to perform a detailed investigation of the relationships between 6 different interleukins and fat distribution using measurements performed by both a dual-energy X-ray absorptiometry (DXA) scan and magnetic resonance imaging (MRI). In addition, we applied a new voxel-based 3D-technique, “Imiomics”, by which interleukin levels were related to each of the > 1 million voxels included in a whole-body MRI scan [27]. A previous study has indicated that relationships between IL-6 and abdominal fat mass were different in men and women [14]. We therefore performed the analysis in the population-based Prospective investigation of Obesity, Energy and Metabolism (POEM) study in males and females separately **(**mean BMI 26–27 kg/m^2^**)**. The hypothesis evaluated was that the six interleukins would be related to fat distribution in different ways.

## 2 Materials and methods

### 2.1 Sample

In the Prospective study on Obesity, Energy, and Metabolism (POEM), 50-year-old men and women were recruited from the general population in the municipality of Uppsala, Sweden. Public population registers were used and invitation was done in a random manner by mail [28]. The participants were invited one month after their 50th birthday. In total, 502 individuals took part in the study. The participation rate was 25%. The study was approved by the Uppsala University ethics committee at (No. 2009/057 and No. 2012/143). Informed consent was given by the participants.

The participants were examined after fasting overnight. The blood samples were drawn between 8 and 10 am. After centrifugation, plasma was frozen in −80C until protein analyses.

Blood pressure was measured in the supine position after 30 min of rest by a mercury sphygmomanometer. Fasting blood glucose and lipids were measured by conventional techniques at the hospital clinical chemistry laboratory.

The participants were asked about their exercise habits, which were graded into four groups; sedentary, only light exercise only (no sweat), exercise at least 0.5 h 1–2 times a week and exercise > 2 times a week.

The circumferences of the waist and hip were measured at the umbilical and trochanter levels, respectively. Using these measurements, waist to hip ratio (WHR) was calculated. Fat and lean mass measurements were established using DXA. Whole-body (head to toe) MRI as well as dedicated imaging of pancreas and liver were performed on subjects who volunteered for this part of the study. The MRI scan was acquired on a separate day, not more than one month from the main study visit. The study in this paper includes a subset of the POEM cohort consisting of the 326 subjects for which the MRI registration was successful [29].

None of the subjects reported any cardiovascular disease. The prevalence of diabetes was low in the sample (1.8% in males and 2.3% in females).

### 2.2 Protein analyses

Using the proximity extension assay (PEA) technique, 92 proteins were measured by the OLINK CVD-1 chip (Olink, Uppsala, Sweden). In the present study, we evaluated the 6 proinflammatory interleukins with a call-rate > 75%. Details on measurements, quality control etc have been given previously [30].

### 2.3 DXA

A Dual-energy X-ray absorptiometry scanner (DXA; Lunar Prodigy, GE Healthcare) was used to measure regional (trunk, leg, arm) and total body fat and lean mass. One experienced nurse performed all scans to minimize potential operator bias. The scans were all performed in the same room. Triple measurements in 15 subjects with repositioning were used to calculate the precision error of the DXA measurements. This approach for computing the precision error follows the recommendations by the International Society for Clinical Densitometry. The precision error was 1.5% and 1.0% for total fat and lean mass, respectively. Automatic edge detection was consistently employed in the analysis. In addition, all scans were carefully checked for errors. Manual correction was performed when necessary.

### 2.4 MRI

A 1.5 T clinical MR system (Philips Achieva, Philips Healthcare, Best, Netherlands) was used to acquire whole-body MRI images of the subjects in supine position. The acquisition was carried out with a continuously moving bed setup using the integrated body coil. A dedicated scan of the liver and the pancreas was also undertaken to enable detailed analysis of liver and pancreas fat content. For additional information about the imaging protocol, see [29], [31], [32].

The liver and pancreas fat quantification was performed by manual delineation of the volume of interest. The software ImageJ (version 1.45 s) was used for the delineation. As much as possible of the volume of interest was demarked by a trained operator. Tissue borders were avoided to limit partial volume effects. As measurements of tissue fat content, the median fat contents were used

Quantification of the VAT and SAT depots were performed by deforming manually defined depots to all other subjects. The deformation was done by utilizing the same image-registration method that was used for the Imiomics analysis, as described below. This process was done separately for male and female. Further processing of the deformed regions (thresholding operations) was done to remove voxels with fat content < 50%.

### 2.5 Imiomics

The Imiomics technique builds on image registration. In image registration, deformation field is computed and applied to deform a target image to match a (fixed) reference image. The deformation field defines a point-to-point correspondence between the reference and target images. These correspondences are used in Imiomics analyses to deform whole-body MRI images to a single reference whole-body MRI volume. Each point in all volume images in the cohort has a corresponding point in the reference coordinate system, i.e., the reference whole-body MRI volume. The voxel-wise statistical analysis procedure [32] is enabled by this pointwise correspondence. In the statistical analysis, associations between fat content from the MRI and tissue volume derived from the deformation field (resulting from the image registration) and non-imaging data can be studied.

It is crucial that the image registration method is reliable in Imiomics analyses, and a tissue-specific handling of bone, lean tissue and adipose tissue is employed. The degree of deformation elasticity tends to differ across these different tissue types, when aligning two images. The tissue specific processing is implemented in the image registration process by performing deformations of the different tissues sequentially and applying tissue-specific registration parameters. In the Imiomics image registration method, the following three steps are carried out: 1) Piece-wise affine articulated registration of bone sections; 2) Water image registration with constraints on bone; 3) Fat images registration with constraints on bone and water. The image registration method has previously been presented and evaluated [33] on MRI water–fat image data.

### 2.6 Statistics

Initially, a correlation matrix based on Pearsońs correlation coefficient was created for the six interleukins.

Thereafter, relationships between traditional measurements of anthropology and fat distribution and the six interleukins was evaluated by linear regression analysis. The relationships are presented as regression coefficients and were stratified by sex. Some variables (those that were skewed to the right - liver fat, pancreatic fat, VAT, SAT) were ln-transformed to achieve a normal distribution. In order to improve the comparison between measurements of anthropology and fat distribution, these were transformed to a standard deviation-scale. The analyses were performed unadjusted, since all subjects were aged 50 years.

STATA 16 was employed for the computations (Stata Corp, College Station, TX, USA).

For the Imiomics analyses, r-value maps were generated for all aprameters by using Spearman non-parametric correlation. MATLAB (version 2016b, The MathWorks Inc., Natick, MA) was used for these calculations. The colormap jet was used to visualize the r-values. The colormap jet represents strong positive correlations (near one) as dark red and strong negative correlations (near minus one) as dark blue.

The Imiomics results were analyzed visually. Only associations corresponding to P-values far below 0.05 visible in many image elements within a certain anatomical structure were reported. This procedure was used to minimize the number of false positives from the large number of tests performed.

## 3 Results

### 3.1 Relationships between the interleukin measurements

**Fig. 1:**
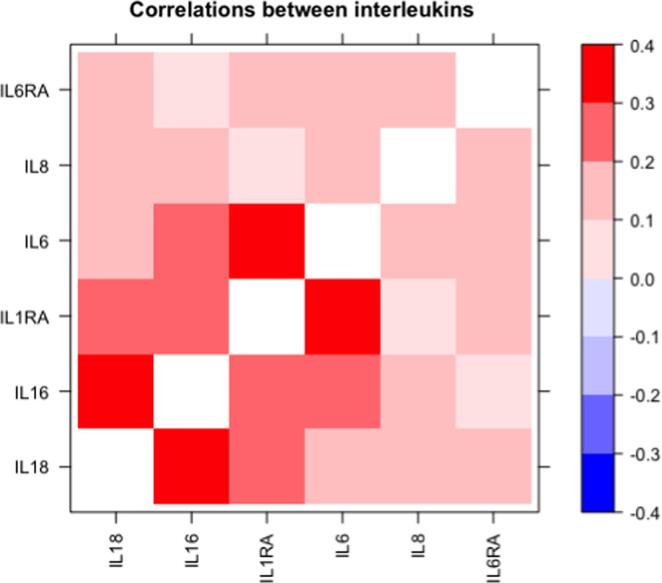
Correlation matrix relating the interleukins to each other. The color-coding is for Pearsonścorrelation coefficient. Correlations > 0.10 corresponds to p < 0.05.

As could be seen in Fig. 1, the relationships between the six interleukin measurements varied from a correlation coefficient of 0.07 to 0.37, with the closest relationships between IL-16 and IL-18 and between IL-6 and IL1RA, while the relationships between IL-8 and IL-1RA and between IL-16 and IL-6R showed p > 0.05.

No major differences in the relationships between the measured plasma interleukins were seen between men and women (see Table 1).

**Table 1:** Traditional measurements of body composition and some basic characteristics in the sample.

	Females		Males	
	N	Mean (SD)	N	Mean (SD)
Height (cm)	165	166.2 (6.6)	156	179.2 (6.3)
Weight (kg)	165	71.8 (12.6)	156	85.7 (11.9)
Waist circumference (cm)	165	89.6 (11.1)	156	94.5 (10)
Hip circumference (cm)	165	103.1 (8.4)	156	101.1 (6.3)
BMI (kg/m^2^)	165	26.0 (4.6)	156	26.7 (3.6)
Waist/hip-ratio	165	0.9 (0.1)	156	0.9 (0.1)
Total fat mass at DXA (kg)	154	26.6 (9.8)	148	21.8 (8.8)
Total lean mass (kg)	154	42.3 (4.7)	148	60.1 (5.9)
Fat mass at trunk (kg)	154	13.5 (5.5)	148	13.5 (5.7)
Fat mass at leg (kg)	154	9.5 (3.4)	148	5.7 (2.3)
Lean mass at leg (kg)	154	13.7 (1.8)	148	20.1 (2.3)
Fat mass at arm (kg)	154	2.8 (1.2)	148	1.9 (0.9)
Lean mass at arm (kg)	154	4.6 (0.7)	148	7.8 (1.1)
Liver fat (%)	138	3.4 (5.7)	121	5 (5.7)
Pancreas fat (%)	138	4.3 (3.0)	120	7.1 (6.4)
Visceral adipose tissue (L)	165	2.5 (1.5)	156	4.4 (2.2)
Subcutaneous adipose tissue (L)	165	7.6 (3.4)	156	5.7 (2.8)
Systolic blood pressure (mmHg)	165	123.4 (17)	156	127.8 (15.9)
Diastolic blood pressure (mmHg)	165	74.2 (9.9)	156	79.1 (10.7)
Fasting glucose (mmol/l)	165	4.5 (0.5)	156	4.6 (0.8)
Serum cholesterol (mmol/l)	165	5.2 (0.9)	156	5.4 (1.1)
LDL- cholesterol (mmol/l)	165	3.3 (0.8)	156	3.5 (0.9)
HDL- cholesterol (mmol/l)	165	1.5 (0.3)	156	1.3 (0.4)
Smoking (%)	165	4.4	156	14.6
Diabetes (%)	165	1.8	156	2.5
Exercise habits	165	Sedentary:10 Light exercise:28 Exercise 1–2/week:35 Exercise > 2/week:27	156	Sedentary:16 Light exercise:18 Exercise 1–2/week:35 Exercise > 2/week:31

### 3.2 Cytokines vs traditional measurements of body composition

As could be seen in Table 2, Table 3, both IL-1RA and IL-6 were related to all indices of body fat, but not to lean mass indices. IL-1RA levels were generally more closely related to the indices of body fat than IL-6, and the relationship between IL-6 and liver fat showed p > 0.05 in both men and women, but when adjusting for sex (instead of stratifying on sex), the relationship between IL-6 and liver fat showed a p-value of 0.00037.

**Table 2:** Relationships between plasma levels of interleukin-1 receptor antagonist (IL-1RA) and traditional measurements of body composition. Both IL-1RA and traditional measurements of body composition were on an SD scale in the calculations to improve comparisons between results. Relationships with p > 0.05 are given in a grey tone.

	**Males**				**Females**			
**Variable**	**Beta**	**95%CI lower**	**95%CI higher**	**p-value**	**Beta**	**95%CI lower**	**95%CI higher**	**p-value**
Height	−0.13	−0.32	0.07	0.21	−0.19	−0.39	0.01	0.06
Weight	0.37	0.22	0.52	1.7e−06	0.31	0.13	0.49	0.0009
Waist circumference	0.42	0.28	0.55	1.0e−09	0.39	0.23	0.54	1.3e−06
Hip circumference	0.35	0.2	0.51	7.7e−06	0.29	0.11	0.47	0.001
BMI	0.42	0.28	0.57	6.9e−09	0.38	0.22	0.55	6.0e−06
Waist/hip-ratio	0.44	0.28	0.59	2.7e−08	0.39	0.22	0.56	7.6e−06
Total fat mass at DXA	0.44	0.3	0.57	1.9e−10	0.43	0.27	0.59	6.0e−08
Total lean mass	−0.03	−0.28	0.21	0.78	−0.19	−0.45	0.07	0.15
Fat mass at trunk	0.4	0.28	0.52	3.9e−11	0.41	0.27	0.55	1.8e−08
Fat mass at leg	0.49	0.3	0.69	6.8e−07	0.45	0.24	0.66	0.00001
Lean mass at leg	−0.02	−0.25	0.21	0.88	−0.14	−0.38	0.1	0.26
Fat mass at arm	0.46	0.31	0.62	9.7e−09	0.44	0.26	0.62	1.1e−06
Lean mass at arm	−0.13	−0.37	0.11	0.30	−0.19	−0.43	0.05	0.12
Liver fat	0.4	0.25	0.55	1.0e−07	0.38	0.21	0.55	0.00001
Pancreas fat	0.25	0.1	0.4	0.0009	0.2	0.04	0.36	0.01
Visceral adipose tissue	0.47	0.33	0.61	4.8e−11	0.45	0.29	0.62	3.9e−08
Subcutaneous adipose tissue	0.35	0.22	0.47	4.0e−08	0.31	0.17	0.46	0.00002

**Table 3:** **Relationships between plasma levels of interleukin-6 (IL-6) and traditional measurements of body composition**. Both IL-6 and traditional measurements of body composition were on an SD scale in the calculations to improve comparisons between results. Relationships with p > 0.05 are given in a grey tone.

	Males				Females			
Variable	Beta	95%CI lower	95%CI higher	p-value	Beta	95%CI lower	95%CI higher	p-value
Height	−0.01	−0.23	0.22	0.96	−0.02	−0.25	0.22	0.88
Weight	0.24	0.06	0.43	0.0082	0.24	0.02	0.46	0.032
Waist circumference	0.23	0.07	0.4	0.0056	0.22	0.02	0.41	0.028
Hip circumference	0.26	0.08	0.44	0.0053	0.24	0.03	0.46	0.023
BMI	0.27	0.1	0.45	0.0024	0.26	0.06	0.46	0.011
Waist/hip-ratio	0.18	−0.01	0.37	0.061	0.14	−0.07	0.35	0.19
Total fat mass at DXA	0.3	0.13	0.47	0.00055	0.3	0.1	0.5	0.0033
Total lean mass	0.02	−0.27	0.3	0.91	−0.05	−0.36	0.26	0.75
Fat mass at trunk	0.28	0.12	0.43	0.00038	0.28	0.1	0.47	0.0022
Fat mass at leg	0.35	0.11	0.59	0.0043	0.32	0.06	0.57	0.016
Lean mass at leg	0.06	−0.21	0.33	0.66	0.01	−0.28	0.3	0.95
Fat mass at arm	0.31	0.11	0.51	0.0023	0.29	0.06	0.51	0.012
Lean mass at arm	−0.08	−0.37	0.2	0.55	−0.12	−0.41	0.17	0.41
Liver fat	0.17	−0.01	0.35	0.057	0.14	−0.07	0.34	0.18
Pancreas fat	0.19	0.03	0.36	0.023	0.17	−0.01	0.34	0.069
Visceral adipose tissue	0.29	0.11	0.46	0.0012	0.28	0.07	0.48	0.0080
Subcutaneous adipose tissue	0.26	0.11	0.41	0.00052	0.26	0.08	0.44	0.0037

Neither IL-6R, nor IL-8 or IL-18, showed any consistent significant relationships vs the traditional measurements of body composition, while IL-16 showed relationships being of borderline significance (p-value 0.02–0.07, suppl tables 1–4) for most of the adipose tissue measurements in the sex-stratified analysis, except for liver fat. These relationships all showed p < 0.05 when adjusting for sex (instead of stratifying on sex).

When deleting the 7 diabetic subjects and adjusting for exercise habits, essentially the same results as reported above were seen.

### 3.3 Cytokines vs Imiomics

IL-1RA

**Fig. 2:**
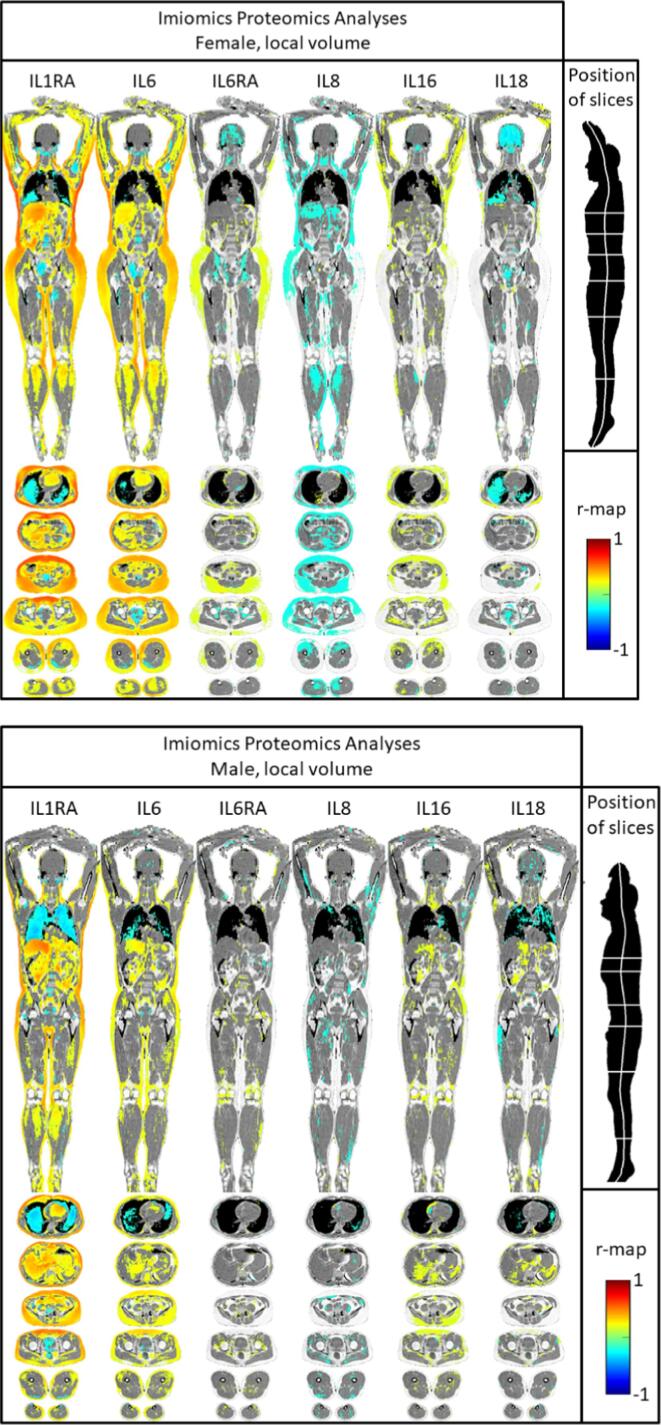
Relationships between 6 different interleukins or receptor antagonists and local volume using voxel-wise analyses in women (upper panel) and men (lower panel). In image elements with P values>0.05, the fat–water signal, weighed so that pure fat is white and pure water is gray, is shown. See also supplementary files S1 and S2.

**Fig. 3:**
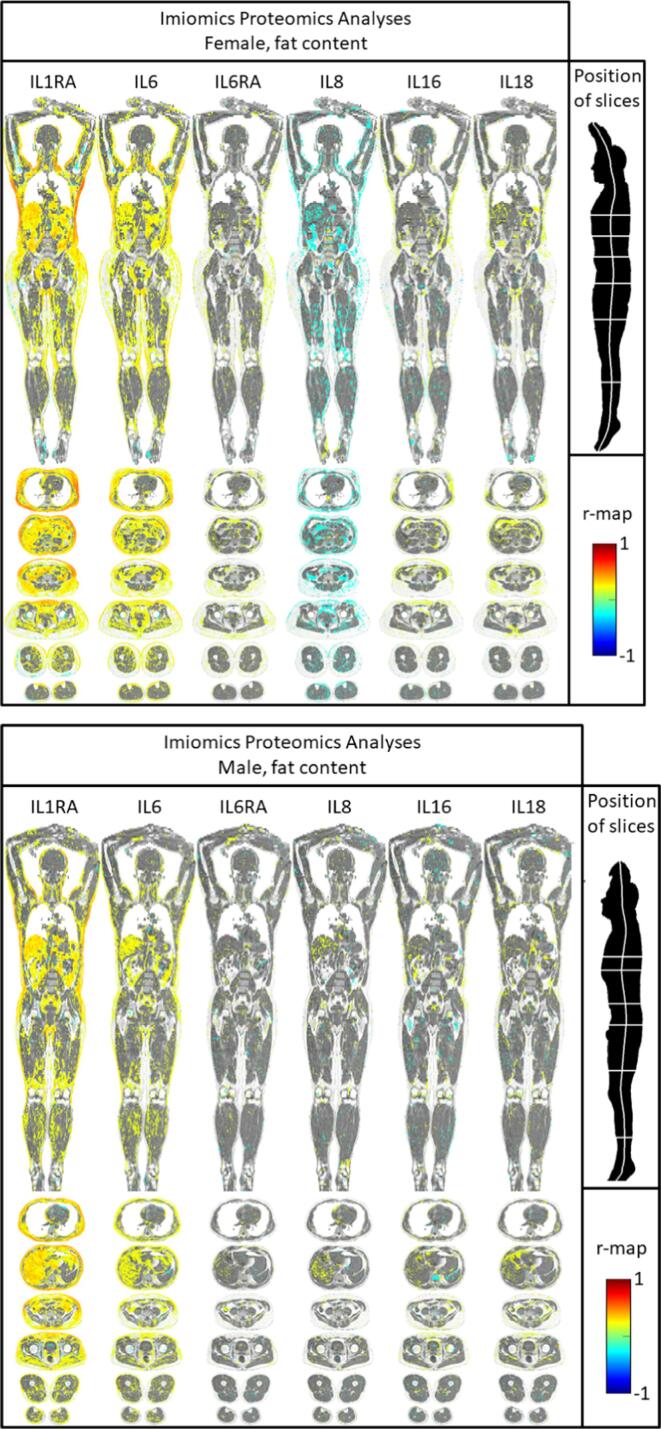
Relationships between 6 different interleukins or receptor antagonists and fat content using voxel-wise analyses in women (upper panel) and men (lower panel). In image elements with P values>0.05, the fat–water signal, weighed so that pure fat is white and pure water is gray, is shown. See also supplementary files S3 and S4.

In both men and women, IL-1RA levels were closely related to expansion of both SAT and VAT, as well as to expansion of liver size and the blood volume in the heart (see Fig. 2). Of notice is that IL-1RA levels were related to expansion of SAT from the arm to the thigh, including the total circumference of the body. IL-1RA levels were also related to a reduction in lung volume in the lower parts of the lungs. IL-1RA levels were also related to the lipid content in the expanded parts described above (Fig. 3).

IL-6

In both men and women, IL-6 levels were related to the size and lipid content of different adipose tissue compartments in a similar fashion as IL-1RA. The relationships were slightly less pronounced for IL6 when compared to IL-1RA.

IL-6RA

In sharp contrast to the findings for IL-6, IL-6RA levels were mainly related to an expansion of SAT in the gluteus area, especially in the dorsal parts, in women. This association was not as strong as for IL-6. In males, not significant relationship between an expansion of SAT and IL6RA levels was seen. The lipid content was not significantly related to IL-6RA.

IL-8

In women, IL-8 levels were related to a reduction of SAT in the upper part of the body, as well as a reduction in skeletal muscle volume in the leg. In males, this was less evident. Also a reduction in lipid content in proportion to IL-8 levels was seen in women, but less so in men.

IL-16

In women, IL-16 levels were weakly related to expansion of SAT in the upper part of the body. In males, this was mainly seen at the umbilical level. The lipid content was not significantly related to IL-16.

IL-18

In women, IL-18 levels were mainly related to a reduction of the lung volume in the lower parts of the lungs. In males, this was less evident. The lipid content was not significantly related to IL-18.

## 4 Discussion and conclusion

The present study showed that plasma levels of IL-1RA and IL-6 were related to SAT in both the upper and lower part of the body, as well as to indices of ectopic fat distribution, such as VAT, liver and pancreatic fat. Neither IL-6R, nor IL-8 or IL-18, showed any consistent significant relationships vs the traditional measurements of body composition, while IL-16 showed relationships being of borderline significance. In women, IL-16 levels were weakly related to expansion of SAT in the upper part of the body, while on the contrary, IL-8 levels were related to a reduction of SAT volume. Thus, different interleukins were related to fat distribution in different ways.

All of the four evaluated interleukins are considered to be proinflammatory, although their actions differ (as reviewed in [34], [35], [36], [37], [38], [39], [40]). IL-6 is secreted from many cells apart from leukocytes, such as adipocytes, osteoblasts, and myocytes. IL-6 is highly proinflammatory causing fever and the acute phase response in inflammation. IL-8 is produced by macrophages, as well as endothelial cells and epithelial cells. This cytokine is an important mediator in the innate immune system response. IL-16 is secreted from lymphocytes and epithelial cells and acts like a chemoattractant for certain immune cells, especially CD4 activated T cells. Also IL-18 can be secreted by several cell types, and modulates both innate and adaptive immunity, including interferon-gamma. Dysregulation of IL-18 has been linked to autoimmune and inflammatory diseases. As could be seen in Fig. 1, plasma levels of these four cytokines are related to each other.

We also included two circulating receptor antagonists, IL-1RA and IL-6RA, in the analysis. Although those by definition are anti-inflammatory, the levels increase in inflammation to balance the receptor ligand, and as could be seen in Fig. 1, positive relationships are seen vs the four evaluated interleukins.

As shown by other investigators [15], [16], [18], we found that IL-6 levels were related to VAT, as well as to liver fat. However, in accordance with findings from the Framingham study [12], the relationship vs SAT was as strong, and both the DXA measurements and the Imiomics analysis showed that the relationship between IL-6 levels and SAT was present both in the upper and lower part of the body. Thus, from this analysis it is evident that IL-6 levels are linked to both an appropriate storage of fat in SAT as well as storage in ectopic tissues, such as VAT and the liver. A similar picture emerged when also another marker of a proinflammatory state, IL-1RA levels, was evaluated. No differences of interest regarding the relationships for IL-1RA and IL-6 vs fat distribution were noted between males and females.

Despite the fact that the other three interleukins and IL-6RA were related to IL-6 levels, no major consistent relationships were seen between levels of these inflammatory markers and fat distribution. In women, IL-16 levels were weakly related to expansion of SAT in the upper part of the body, while on the contrary, IL-8 levels were related to a reduction of SAT volume. Thus, it is evident that different proinflammatory cytokines are not related to fat distribution in a uniform manner.

Plasma IL-1RA and IL-6 levels were also related to an increased blood volume in the heart. The latter is an indicator of myocardial dilatation, and since obesity will increase the blood volume by increased tubular reabsorption of sodium and water [41], it is likely that IL-1RA and IL-6 levels were related to blood volume in the heart by this mechanism.

Generally, the findings using the traditional measurements were mirrored in the Imiomics analysis. The advantage with Imiomics is that it apart from visualizing the findings in a comprehensive and intuitive fashion also provides more information on anatomical details, as well as giving relationships for both volume and lipid content of each voxel. For example, the levels of IL1RA is related to SAT in general, but as could be seen in Fig. 2, the closest correlation was seen vs the parts of SAT being located on the ventral side just above the pubic bone. This is of interest since we previously have found the metabolic syndrome to be closely related to SAT in this location as well [27].

In general, the findings, using both traditional measurements and the Imiomics technique were very similar in both sexes regarding the relationships vs IL6 and IL1RA. For the other interleukins, we found some sex-differences that did not displayed any consistent patterns. It could only be speculated upon if those findings are due to differences in body composition between men and women or if they are merely chance findings.

Despite a similar BMI, the women show a higher total fat mass compared to men. The women also have a proportionally higher waist circumference compared to the definition of abdominal obesity compared to men. These differences between the sexes in this sample might explain some of the sex-differences regarding the relationships between the traditional body composition measurements and the interleukin levels.

A negative association between some of the interleukins and lung volume was noted. This is probably due to the fact that abdominal obesity forces the diaphragm cranially and thereby compress the lower parts of the lungs so that the lung volumes, especially in the lower parts are reduced. Since especially IL6 and IL1RA levels were related to VAT and liver volume this is likely the explanation why those levels also are inversely related to lung volume.

We did find that IL6RA and IL18 levels were associated with a decrease in volume in the head area in the female group. This is likely to be a chance finding in this study in which the MRI scanning was not optimized for brain volume measurements, since we did not find any tendency for relationships between IL6RA and IL18 levels and brain volume in another sample (the PIVUS study), in which dedicated brain MRI scans were performed and brain volume was evaluated by a dedicated software (p = 0.89 vs IL-18 and p = 0.56 vs IL1RA) [42].

The major strength of the present study is that we measured fat distribution by both traditional measurements, DXA and MRI, but also by the novel voxel-based Imiomics method, which gives a detailed 3D view of relationships between interleukin levels and body composition.

In the present study, six interleukins (or their receptor antagonists) were chosen from the prespecified CVD-1 chip manufactured by OLINK. If would have been desirable to evaluate many more interleukins, like IL-1 beta, IL-33, IL-15, IL-17 or IL-10, but these other interleukins were unfortunately not included amongst the available proteins.

A limitation is that our sample consists of Swedish individuals all aged 50, so the results have to be confirmed in other samples with other geographical, age and ethnical characteristics.

In conclusion, of six evaluated interleukins, plasma IL-1RA and IL-6 levels were related to adipose tissue in all parts of the body, while a diverse picture was seen for other interleukins, suggesting that different interleukins are related to fat distribution in different ways.

## CRediT authorship contribution statement

**Robin Strand:** Conceptualization, Data curation, Formal analysis, Funding acquisition, Investigation, Methodology, Project administration, Software, Validation, Visualization, Writing - original draft, Writing - review & editing. **Joel Kullberg:** Conceptualization, Data curation, Formal analysis, Funding acquisition, Investigation, Methodology, Project administration, Resources, Software, Validation, Visualization, Writing - original draft, Writing - review & editing. **Håkan Ahlström:** Conceptualization, Data curation, Funding acquisition, Methodology, Project administration, Resources, Supervision, Writing - original draft, Writing - review & editing. **Lars Lind:** Conceptualization, Data curation, Formal analysis, Funding acquisition, Investigation, Methodology, Project administration, Resources, Supervision, Validation, Visualization, Writing - original draft, Writing - review & editing.

## Declaration of Competing Interest

Joel Kullberg and Håkan Ahlström are cofounders, co-owners of and part time employees of Antaros Medical AB, BioVenture Hub, Mölndal, Sweden. A patent application, P1318PC00, by Robin Strand, Joel Kullberg and Håkan Ahlström describing the image registration method used in this manuscript is currently under review. Antaros Medical is currently holding the rights to the patent application.
