# Supplementary material for: Relationships between plasma levels and six proinflammatory interleukins and body composition using a new magnetic resonance imaging voxel-based technique
Source: Cytokine X. 2020 Dec 21;3(1):100050. doi: 10.1016/j.cytox.2020.100050 (PMC7885882; doi:10.1016/j.cytox.2020.100050)
Supplement: Supplementary data 5 [file mmc5.docx]

S1: Fly-through video of Imiomics correlation maps showing relationships between 6 different interleukins or receptor antagonists and local volume using voxel-wise analyses in women. In image elements with P values greater than .05, the fat-water signal, weighed so that pure fat is white and pure water is gray, is shown. Upper row from left to right: IL1RA, IL6, IL6RA. Bottom row from left to right: IL8, IL16, IL18. See also Figure 2.

S2: Fly-through video of Imiomics correlation maps showing relationships between 6 different interleukins or receptor antagonists and local volume using voxel-wise analyses in men. In image elements with P values greater than .05, the fat-water signal, weighed so that pure fat is white and pure water is gray, is shown. Upper row from left to right: IL1RA, IL6, IL6RA. Bottom row from left to right: IL8, IL16, IL18. See also Figure 2.

S3: Fly-through video of Imiomics correlation maps showing relationships between 6 different interleukins or receptor antagonists and fat content using voxel-wise analyses in women. In image elements with P values greater than .05, the fat-water signal, weighed so that pure fat is white and pure water is gray, is shown. Upper row from left to right: IL1RA, IL6, IL6RA. Bottom row from left to right: IL8, IL16, IL18. See also Figure 3.

S4: Fly-through video of Imiomics correlation maps showing relationships between 6 different interleukins or receptor antagonists and fat content using voxel-wise analyses in men. In image elements with P values greater than .05, the fat-water signal, weighed so that pure fat is white and pure water is gray, is shown. Upper row from left to right: IL1RA, IL6, IL6RA. Bottom row from left to right: IL8, IL16, IL18. See also Figure 3.
